# A real-world study on characteristics, treatments and outcomes in US patients with advanced stage ovarian cancer

**DOI:** 10.1186/s13048-020-00691-y

**Published:** 2020-08-31

**Authors:** Daniel C. Beachler, Francois-Xavier Lamy, Leo Russo, Devon H. Taylor, Jade Dinh, Ruihua Yin, Aziza Jamal-Allial, Samuel Dychter, Stephan Lanes, Patrice Verpillat

**Affiliations:** 1 Safety and Epidemiology, HealthCore, Inc, 123 Justison Street, Suite 200, Wilmington, DE 19801 USA; 2 Global Epidemiology, Merck KGaA, Darmstadt, Germany; 3 Global Medical Epidemiology, Pfizer Inc, Collegeville, PA USA; 4 Ingenio Rx, Anthem Inc, Andover, MA USA; 5 Global Product Development, Pfizer Inc, La Jolla, CA USA

**Keywords:** Epidemiology, Ovarian Cancer, Advanced stage, Treatment patterns, Health outcome of interest, Survival

## Abstract

**Background:**

Detailed epidemiologic descriptions of large populations of advanced stage ovarian cancer patients have been lacking to date. This study aimed to describe the patient characteristics, treatment patterns, survival, and incidence rates of health outcomes of interest (HOI) in a large cohort of advanced stage ovarian cancer patients in the United States (US).

**Methods:**

This cohort study identified incident advanced stage (III/IV) ovarian cancer patients in the US diagnosed from 2010 to 2018 in the HealthCore Integrated Research Database (HIRD) using a validated predictive model algorithm. Descriptive characteristics were presented overall and by treatment line. The incidence rates and 95% confidence intervals for pre-specified HOIs were evaluated after advanced stage diagnosis. Overall survival, time to treatment discontinuation or death (TTD), and time to next treatment or death (TTNT) were defined using treatment information in claims and linkage with the National Death Index.

**Results:**

We identified 12,659 patients with incident advanced stage ovarian cancer during the study period. Most patients undergoing treatment received platinum agents (75%) and/or taxanes (70%). The most common HOIs (> 24 per 100 person-years) included abdominal pain, nausea and vomiting, anemia, and serious infections. The median overall survival from diagnosis was 4.5 years, while approximately half of the treated cohort had a first-line time to treatment discontinuation or death (TTD) within the first 4 months, and a time to next treatment or death (TTNT) from first to second-line of about 6 months.

**Conclusions:**

This study describes commercially insured US patients with advanced stage ovarian cancer from 2010 to 2018, and observed diverse treatment patterns, incidence of numerous HOIs, and limited survival in this population.

## Background

Ovarian cancer is the most lethal gynecologic malignancy [1] and the fifth most common cause of cancer death for women in the United States (US) [1]. Epithelial ovarian cancer is primarily treated with surgery and platinum-based chemotherapy, and can also be treated with radiation, hormone, or targeted therapy. Many new treatments, including poly ADP-ribose polymerase (PARP) inhibitors, are indicated specifically for advanced stage ovarian cancer, [2] while potential new therapies, such as immunotherapies, are being investigated [3].

Randomized trials have suggested that adverse events including hypertension, neutropenia, liver-related toxicity, fatigue, anemia and diarrhea can occur commonly after initiation of certain ovarian cancer therapies, [4–6] but less is known about the incidence and types of health outcomes of interest (HOIs) occurring in the general ovarian cancer population. Randomized trials are tightly controlled studies that commonly use small and narrowly defined populations. Recent publications have suggested that trial populations are significantly younger, have higher income, and have fewer co-morbidities than the general cancer population [7–9].

Real world evidence on the characteristics, treatment patterns, incidence of HOIs, and outcomes (including survival) of advanced stage ovarian cancer patients has been limited [10], partially due to the lack of specific cancer information, such as the stage of disease, in large administrative claims databases. Recently, we developed a validated algorithm to define advanced stage ovarian cancer using supervised machine learning techniques [11]. In this study, we applied this algorithm to an administrative claims database to identify a large cohort of advanced stage ovarian cancer patients and described their characteristics, treatment patterns, survival, and incidence rates of HOIs that could be utilized as comparator incidence rates for new and future ovarian cancer therapies indicated for advanced stage ovarian cancer.

## Methods

### Population and design

This study included incident advanced stage ovarian cancer patients in the US using the HealthCore Integrated Research Database (HIRD). The HIRD is a longitudinal medical and pharmacy claims database from health plan members across each region of the US. Member enrollment, medical care, outpatient prescription drug use, outpatient laboratory test result data, and health care utilization are tracked for health plan members.

Claims databases lack certain types of clinical information not needed for billing purposes, such as cancer stage. To overcome this limitation, we linked claims data with three state cancer registries (Ohio, Kentucky, and New York) and the HealthCore Integrated Research Environment (HIRE) Oncology data. HIRE Oncology is a pre-authorization program in which clinical data is obtained through physicians’ submissions of intentions to use certain cancer treatments, and has shown good agreement with medical records with regard to cancer stage [12]. Advanced stage was defined in the registries and HIRE Oncology as epithelial ovarian cancer, either locally advanced (Stage IIIa, IIIb or IIIc) or metastatic (Stage IV). Subsequently, we developed a claims-based predictive model algorithm for advanced stage ovarian cancer among the subset of patients with clinical data using least absolute shrinkage and selection operator (lasso) regression and 20-fold cross validation [11]. The predictive model for advanced stage (III or IV) had a high PPV (95%), specificity (90%), and sensitivity (70%) when validated using data from the state cancer registries and HIRE Oncology, using an 80% probability threshold for defining a case [11].

To identify patients with confirmed incident advanced stage ovarian cancer, patients needed to meet the following inclusion criteria: at least one diagnosis code in any claims position for ovarian cancer (codes starting with International Classification of Diseases [ICD]-9: 1830 or ICD-10: C56; Supplemental Table 1) in the HIRD between January 1, 2010 and January 31, 2018, continuously enrolled in a health plan captured by the HIRD for at least 6 months prior to the first ovarian cancer diagnosis (to restrict to newly diagnosed (incident) cases), and identified as an advanced cancer patient by either matching to a cancer registry, HIRE Oncology, [11, 12] or meeting the predictive algorithm for advanced disease [11].

Follow-up for this cohort of advanced stage ovarian cancer was identical to the inclusion period (January 2010 to January 2018). For each patient, the predictive probability of advanced stage ovarian cancer was computed each time a patient had a claim in the predictive model (hypothetical example of a patient results in Supplemental Fig. 1, Supplemental Table 2). The date of incident advanced cancer (i.e. the index date for the start of follow-up) was defined as the first date the patient met the advanced stage predictive model’s probability threshold of 80% or higher (Supplemental Fig. 1, Supplemental Table 2). The date of incident advanced cancer defined by the predictive model was within 1 month of cancer registry date for 84% of the patients and the median difference between the registry and model was 1 day apart. For patients with confirmed advanced disease who did not meet the predictive model algorithm, we used the cancer registry or HIRE Oncology date as the date of incident advanced cancer. Cases are defined as “advanced stage at diagnosis” if their advanced stage date (from cancer registry, HIRE Oncology, or predictive model) was within 1 month of their first cancer diagnosis in claims, otherwise they are defined as “Diagnosed as early stage and progressed to advanced stage”.

Follow-up started for an individual at the advanced stage index date and continued until they were censored (either by death, end of health plan enrollment, or end of study period (January 2018)). We did not require a set amount of person-time after the advance stage index date, thus a subset of patients in this cohort died or lost to follow-up soon after the advanced stage index date.

Patients were described in terms of demographic and clinical characteristics, prior and concomitant treatments, key incident HOIs, lines of treatment, and mortality. Selected characteristics were presented stratified by treatment line, which was inferred based on observed patterns of medication use which included assumptions such as 28-day cycles and a new line occurring when there were more than 60 days between two cycles or if there were treatment switches or a treatment added. We also identified the 25 most frequently dispensed medication classes during the 12 months before the advanced stage ovarian cancer index date and separately for the 12 months after the advanced stage ovarian cancer index date. The medication classes were defined at the four-digit Generic Product Identifier (GPI) level. Diagnoses are not linked to a specific prescription, and thus some of the record treatments may have been specified for other cancers such as breast cancer, if a patient had multiple malignancies.

We described characteristics for patients who were platinum therapy sensitive, platinum resistant, or platinum refractory, which were defined similarly to previously published studies [13, 14]. The categorization was defined using medication dispensing data for platinum sensitive agents (cisplatin, carboplatin, or oxaliplatin) and other chemotherapies, and time until use of a second-line therapy.

We linked claims to the US National Death Index (NDI) to identify mortality outcomes and cause of death, following NDI standards for identification of death [15]. We also evaluated two real-world surrogates of cancer progression in this cohort, time to treatment discontinuation or death (TTD), and time to next treatment or death (TTNT) [16]. We defined TTD as the time from the date of initiation of a first-line systemic anti-cancer therapy after the advanced stage index date to the earliest of discontinuation (> 60 days without first-line treatment; event), death (event) or loss to follow-up in the HIRD (administrative censor, not an event). TTNT was defined as the time from the date of the first-line treatment after the advanced stage index date to the earliest of a second-line treatment (event), death (event), or loss to follow-up in the HIRD (administrative censor, not an event). We restricted mortality, TTD, and TTNT analyses to the patients available for linkage to the NDI, as a subset of the cohort was unable to be linked due to privacy restrictions. This study was approved by the New England Institutional Review Board (Work Order Number 1–9472-1).

### Statistical analysis

Patient characteristics and treatments received were described by counts and percentages for categorical variables and statistics such as mean, standard deviation (SD), and median for continuous variables. Person-time incidence rates and Poisson 95% confidence intervals (CIs) were calculated for pre-specified HOIs. These pre-specified HOIs were identified with attention to the Medical Dictionary for Regulatory Activities (MedDRA) classification system and FDA approved standardized case definitions, when possible. MedDRA is not always directly translatable to use in administrative claims data but can sometimes be approximated with ICD codes. These HOIs required two or more ICD-9/ICD-10 diagnosis codes in any setting or at least one ICD-9/ICD-10 diagnosis code in the inpatient setting (codes available upon request). For the main analysis, the incidence rate of each HOI was determined from the case definition date for advanced ovarian cancer (index date) through the first HOI of a given type, or the end of the patient’s follow-up due to a censoring event, whichever is sooner. Incidence rates of HOIs after systemic anticancer therapy (while with advanced stage disease) were also conducted. We also assessed severe HOIs as those requiring hospitalization or ER visit as defined by the primary diagnosis on the facility claim.

Administrative claims-based assessments of disease incidence can be inaccurate for repeated events, as it is not always possible to distinguish between a patient who has a past medical history of a condition and one who has been newly diagnosed or experienced an acute event. For this reason, for most HOIs, patients were followed from cohort entry (or treatment initiation from some analyses) until their first recorded event of a given type, and then censored from follow-up for that event type. Unless otherwise specified, we excluded patients who presented the HOI prior to start of study follow-up (i.e. prevalent cases during the baseline period) from these HOI analyses.

The product-limit estimator was used to describe median values and rates of mortality, TTD, and TTNT at one, three, and 5 years and the corresponding Kaplan-Meier curves [17]. In a sensitivity analysis, we also evaluated the rates of mortality when excluding the last 6 months of data provided from the NDI (July 1, 2017 to December 31, 2017) given prior evidence of lower sensitivity of newly released data [15].

## Results

### Descriptive characteristics

We identified 12,659 advanced ovarian cancer patients that met the eligibility criteria for this cohort. Most patients were classified as incident advanced stage at diagnosis (96.7%) rather than incident early stage cancers that progressed to an advanced stage (3.3%) which may often represent recurrent cases. At the time of advanced stage, these patients had a mean (±SD) age of 62 ± 14 years, and 50% were followed after their advanced cancer date for over 17.3 months (Table 1). The comorbidity burden was elevated with a median Deyo-Charlson Comorbidity Index (DCI) score of 6 [18]. The most frequently dispensed medication class in the 12 months before and after the advanced stage index date was opioid combinations (pre: 41.6%; post: 46.3%; Supplemental Table 3). Medication use appeared to increase after the advanced stage index date particular for 5-HT3 receptor agonists (pre: 19.7%; post: 37.1%) and phenothiazines (pre: 13.8%; post: 27.0%) which are both often used to treat nausea (Supplemental Table 3).

**Table 1:** Advanced stage ovarian cancer cohort, demographic characteristics and healthcare utilization (*N*=12,659)

Characteristics	N (%)
Total advanced incident cancer cases from January 1, 2010 to January 31, 2018 (N, %)^a,b^	
Advanced stage at diagnosis, N (%)^c^	12,237 (96.67%)
Diagnosed as early stage and progressed to advanced stage, N (%)	422 (3.33%)
Age (years)	
Mean (SD)	61.94 (14)
Q1	53
Median	62
Q3	72
Region	
Midwest	3436 (28.48%)
Northeast	2293 (19.01%)
South	2976 (24.67%)
West	3358 (27.84%)
Year of index date	
2010-2011	4712 (37.22%)
2012-2014	4468 (35.30%)
2015-2018	3479 (27.48%)
Plan type	
Commercial	9288 (73.37%)
Medicare Advantage	1511 (11.94%)
Medicare, Other	1860 (14.69%)
Duration of health plan membership prior to advanced stage index date (months)	
Mean (SD)	48.16 (28.25)
Q1	23.24
Median	48.20
Q3	68.86
Duration of follow-up (months)	
Mean (SD)	24.52 (23.28)
Q1	6.47
Median	17.25
Q3	35.19
Deyo-Charlson Comorbidity Index (DCI) during follow-up	
Mean (SD)	6.34 (2.63)
Q1	5
Median	6
Q3	8
Number of drugs dispensed during follow-up	
Mean (SD)	12.61 (9.76)
Q1	5
Median	11
Q3	18

*Abbreviations*: *N* Number, *SD* Standard deviation, *US* United States, *Q* Quartile, *ED* Emergency department.

^a^The cohort includes patients who had at least one ICD-9-CM or ICD-10 diagnosis code for ovarian cancer, were continuously enrolled in a health plan contributing data to the HIRD for at least six months, and were confirmed to have advanced ovarian cancer based on staging information from either a cancer registry or the HIRE Oncology data or met the predictive model algorithm for advanced stage ovarian cancer.

^b^Incident cases are individuals for whom at least six months of data were available in the HIRD prior to the first diagnosis of ovarian cancer in claims.

^c^Cases are defined as "advanced stage at diagnosis" if their advanced stage date (from cancer registry, HIRE Oncology, or predictive model) was within one month of their first cancer diagnosis in claims, otherwise they are defined as "Diagnosed as early stage and progressed to advanced stage".

Regarding the treatment for ovarian cancer, close to half of advanced ovarian cancer patients had at least one ovarian cancer-related surgery during follow-up (i.e. after the advanced stage index date) (40.5%), primarily palliative surgery for relief of small bowel obstruction (34.9%; Supplemental Table 3). More than two-thirds received radiotherapy or systemic anti-cancer therapy (68.5%) after the advanced stage index date, the most common being platinum agents (75.3%; carboplatin = 66.3%, cisplatin = 14.1%, and oxaliplatin = 4.4% of treated patients) and taxanes (70.0%; paclitaxel = 64.2% and docetaxel = 12.8% of treated patients). Common specific agents used were carboplatin (66.3%) and paclitaxel (64.2%). There were 68.5% of patients for whom we observed a first line of treatment (including systemic therapy and radiotherapy), 43.9% had a second line, 30.5% had a third line, and 20.5% had four or more lines (Supplemental Table 4). Following first line therapy, there were 12.1% categorized as platinum sensitive, 15.3% as platinum resistant, and 41.7% as platinum refractory (Supplemental Table 4). The age, DCI, and treatment use were largely similar between platinum sensitive and platinum refractory/resistant patients (results available upon request.

Systemic anti-cancer medication class use differed by treatment line (Table 2). The majority of patients were taking platinum and taxane agents in the first treatment line, while the use of angiogenesis inhibitors, hormonal and related agents, antineoplastic antibodies, and antineoplastic antibiotics all became more widely used in later treatment lines (> 25% in the fourth line or higher; Table 2). The most commonly used agents, carboplatin and paclitaxel, were most frequently used in the first treatment line, and the proportion of patients using them were lower in the subsequent treatment lines (~ 50% in first line vs. < 37% in all subsequent treatment lines; Table 2). There were 12% of patients who had a breast cancer diagnosis (in addition to their ovarian cancer diagnosis) noted during their first treatment line therapy, suggesting a small subset of first line therapies may have been for breast cancer.

**Table 2:** Medication classes by treatment line among those receiving anti-cancer treatment in the advanced stage ovarian cancer cohort (*n*=8,325)

	N (%)	N (%)	N (%)	N (%)	N (%)
	All Lines (All treated patients)	First Line	Second Line	Third Line	Fourth Line or Higher
Total cancer treatment episodes with treatment lines observed, among incident cases, N (%)^a,b,c,d^	8325 (100%)	8325 (100%)	5335 (64.1%)	3711 (44.6%)	2487 (29.9%)
**Alkylating Agents**	5863 (70.43%)	5071 (60.91%)	2425 (45.45%)	1230 (33.14%)	1074 (43.18%)
Carboplatin (platinum agent)	5055 (60.72%)	4243 (50.97%)	1926 (36.10%)	890 (23.98%)	819 (32.93%)
Cisplatin (platinum agent)	1075 (12.91%)	546 (6.56%)	368 (6.90%)	257 (6.93%)	258 (10.37%)
Cyclophosphamide	358 (4.30%)	122 (1.47%)	77 (1.44%)	68 (1.83%)	160 (6.43%)
Ifosfamide/Mesna	44 (0.53%)	15 (0.18%)	14 (0.26%)	10 (0.27%)	17 (0.68%)
Ifosfamide	39 (0.47%)	13 (0.16%)	13 (0.24%)	10 (0.27%)	16 (0.64%)
Altretamine	17 (0.20%)	≤10	≤10	≤10	12 (0.48%)
Melphalan	≤10	0 (0%)	0 (0%)	≤10	≤10
Oxaliplatin (platinum agent)	327 (3.93%)	207 (2.49%)	117 (2.19%)	65 (1.75%)	72 (2.90%)
**Mitotic Inhibitors**	5368 (64.48%)	4584 (55.06%)	2033 (38.11%)	975 (26.27%)	904 (36.35%)
Paclitaxel (Taxane)	4892 (58.76%)	4160 (49.97%)	1734 (32.50%)	807 (21.75%)	715 (28.75%)
Albumin bound paclitaxel	3506 (42.11%)	2888 (34.69%)	1182 (22.16%)	540 (14.55%)	543 (21.83%)
Docetaxel (Taxane)	864 (10.38%)	533 (6.40%)	310 (5.81%)	155 (4.18%)	185 (7.44%)
Vinorelbine	123 (1.48%)	18 (0.22%)	30 (0.56%)	31 (0.84%)	80 (3.22%)
**Antimetabolites**	2045 (24.56%)	799 (9.60%)	737 (13.81%)	558 (15.04%)	894 (35.95%)
Gemcitabine	1416 (17.01%)	363 (4.36%)	413 (7.74%)	345 (9.30%)	651 (26.18%)
Capecitabine	219 (2.63%)	86 (1.03%)	87 (1.63%)	55 (1.48%)	89 (3.58%)
Pemetrexed	247 (2.97%)	46 (0.55%)	39 (0.73%)	41 (1.10%)	164 (6.59%)
**Antineoplastic - Angiogenesis Inhibitors**	1572 (18.88%)	524 (6.29%)	482 (9.03%)	429 (11.56%)	716 (28.79%)
Bevacizumab	1567 (18.82%)	524 (6.29%)	481 (9.02%)	427 (11.51%)	712 (28.63%)
**Antineoplastic - Hormonal and Related Agents**	3102 (37.26%)	2095 (25.17%)	1679 (31.47%)	1146 (30.88%)	1148 (46.16%)
Anastrozole	437 (5.25%)	177 (2.13%)	170 (3.19%)	111 (2.99%)	176 (7.08%)
Letrozole	354 (4.25%)	103 (1.24%)	130 (2.44%)	97 (2.61%)	173 (6.96%)
Exemestane	127 (1.53%)	41 (0.49%)	45 (0.84%)	43 (1.16%)	49 (1.97%)
Leuprolide	45 (0.54%)	20 (0.24%)	17 (0.32%)	15 (0.40%)	23 (0.92%)
Goserelin	10 (0.12%)	≤10	≤10	≤10	≤10
Triptorelin	≤10	0 (0%)	0 (0%)	0 (0%)	≤10
Tamoxifen	425 (5.11%)	167 (2.01%)	150 (2.81%)	98 (2.64%)	153 (6.15%)
**Antineoplastics Misc.**	≤10	≤10	≤10	≤10	≤10
**Topoisomerase I/II Inhibitors**	1024 (12.30%)	292 (3.51%)	284 (5.32%)	257 (6.93%)	527 (21.19%)
Topotecan	691 (8.30%)	104 (1.25%)	162 (3.04%)	179 (4.82%)	406 (16.32%)
Irinotecan	220 (2.64%)	93 (1.12%)	78 (1.46%)	58 (1.56%)	99 (3.98%)
Etoposide	184 (2.21%)	95 (1.14%)	46 (0.86%)	20 (0.54%)	61 (2.45%)
**Antineoplastic - Antibodies**	1684 (20.23%)	608 (7.30%)	537 (10.07%)	464 (12.50%)	744 (29.92%)
**Antineoplastic Combinations**	≤10	0 (0%)	≤10	≤10	≤10
**Chemotherapy Rescue/Antidote Agents**	340 (4.08%)	242 (2.91%)	159 (2.98%)	90 (2.43%)	91 (3.66%)
Leucovorin	328 (3.94%)	239 (2.87%)	155 (2.91%)	87 (2.34%)	85 (3.42%)
**Somatostatin**	83 (1%)	28 (0.34%)	24 (0.45%)	26 (0.70%)	32 (1.29%)
**Doxorubicin (Anthracycline)**	1789 (21.49%)	418 (5.02%)	536 (10.05%)	485 (13.07%)	726 (29.19%)
**Antineoplastic Antibiotics**	1848 (22.20%)	468 (5.62%)	548 (10.27%)	488 (13.15%)	735 (29.55%)
**Antineoplastic - Hedgehog Pathway Inhibitors**	0 (0%)	0 (0%)	0 (0%)	0 (0%)	0 (0%)
**Antineoplastic – Immunomodulators**	≤10	≤10	0 (0%)	0 (0%)	0 (0%)
**Antineoplastic Radiopharmaceuticals**	0 (0%)	0 (0%)	0 (0%)	0 (0%)	0 (0%)
**Chemotherapy Adjuncts**	0 (0%)	0 (0%)	0 (0%)	0 (0%)	0 (0%)
**Immune-Checkpoint Inhibitors**	63 (0.76%)	15 (0.18%)	16 (0.30%)	11 (0.30%)	35 (1.41%)
Nivolumab	35 (0.42%)	≤10	12 (0.22%)	≤10	19 (0.76%)
Pembrolizumab	21 (0.25%)	≤10	≤10	≤10	15 (0.60%)
Atezolizumab	0 (0%)	0 (0%)	0 (0%)	0 (0%)	0 (0%)
Avelumab	0 (0%)	0 (0%)	0 (0%)	0 (0%)	0 (0%)
Ipilimumab	11 (0.13%)	≤10	≤10	≤10	≤10
Durvalumab	0 (0%)	0 (0%)	0 (0%)	0 (0%)	0 (0%)
**Antineoplastic Enzyme Inhibitors/PARP inhibitors**	572 (6.87%)	274 (3.29%)	196 (3.67%)	120 (3.23%)	209 (8.40%)
Olaparib	83 (1%)	≤10	9 (0.17%)	12 (0.32%)	66 (2.65%)
Rucaparib	23 (0.28%)	0 (0%)	≤10	≤10	21 (0.84%)
Niraparib	0 (0%)	0 (0%)	0 (0%)	0 (0%)	0 (0%)
Pazopanib	32 (0.38%)	≤10	≤10	≤10	13 (0.52%)

*Abbreviations*: *N* Number, *Misc*. Miscellaneous

^a^ The cohort includes patients who had at least one ICD-9-CM or ICD-10 diagnosis code for ovarian cancer, were continuously enrolled in a health plan contributing data to the HIRD for at least six months, and were confirmed to have advanced ovarian cancer based on staging information from either a cancer registry or the HIRE Oncology data or met the predictive model algorithm for advanced stage ovarian cancer. Patients may have received multiple treatments during each treatment line and may have received cancer treatment prior to line 1 if they were dispensed prior to the advanced stage ovarian index date. Diagnoses are not linked to a specific prescription, and thus some of the record treatments may have been specified for other cancers, if a patient had multiple malignancies.

^b^Incident cases are individuals for whom at least six months of data were available in the HIRD prior to the first diagnosis of ovarian cancer in claims.

^c^Stratum percentages based on total number of cases for all lines.

^d^Patients with follow-up periods shorter than 28 days (specified segment length) were excluded from the treatment line related analyses.

### Health outcomes of interest (HOIs)

The most common pre-defined HOIs among advanced stage ovarian cancer patients included abdominal pain, nausea and vomiting, anemia, and serious infections (each > 24 per 100 person-years; Table 3). Advanced stage ovarian cancer patients also frequently developed malaise/fatigue, hypertension, constipation, pain in joints or limbs, and renal failure (each > 10 per 100-person years; Table 3). Endocrinopathies and immune/autoimmune related event rates were less frequent (e.g., colitis: 3.1 per 100 person-years, type 1 diabetes: 0.5 per 100 person-years; Table 3).

**Table 3:** Incidence rates of health outcomes of interest among advanced stage ovarian cancer patients

Health outcome of interest	After advanced stage date (***n***=12,659)					After anti-cancer therapy date (***n***=7,723)				
	Events	Person-years	IR^a^	95% CI		Events	Person-years	IR	95% CI	
Serious infection^b^	6662	25868	25.78	25.17	26.41	5,862	15938	41.42	40.36	42.49
Rash										
Any rash	686	22684	3.02	2.8	3.26	431	14095	3.06	2.78	3.36
Severe cutaneous rash safety events	87	25534	0.34	0.27	0.42	62	15752	0.39	0.3	0.5
Colitis	725	23251	3.12	2.9	3.35	464	14567	3.19	2.91	3.49
Pneumonitis										
Interstitial lung disease	119	25622	0.46	0.39	0.55	62	15812	0.39	0.3	0.5
Hypersensitivity pneumonitis	≤10	n/a	0.03	0.02	0.06	≤10	n/a	0.03	0.01	0.06
Pneumonitis or acute interstitial pneumonitis	137	25653	0.53	0.45	0.63	83	15824	0.52	0.42	0.65
Hepatitis										
Hepatic failure	764	23057	3.31	3.08	3.55	510	14112	3.61	3.31	3.94
Autoimmune hepatitis	≤10	n/a	0.02	0.01	0.05	≤10	n/a	0.03	0.01	0.07
Hepatitis (not specified as viral)	66	25559	0.26	0.2	0.33	23	15798	0.15	0.09	0.21
Liver disorder	1439	21567	6.67	6.33	7.02	960	13128	7.31	6.86	7.79
Transaminases increased	272	25086	1.08	0.96	1.22	169	15483	1.09	0.94	1.27
Nephritis	1966	21201	9.27	8.87	9.69	1,309	13361	9.80	9.28	10.34
Renal failure	2057	21425	9.60	9.19	10.02	1,379	13524	10.20	9.67	10.75
Endocrinopathies										
Adrenal insufficiency	87	25608	0.34	0.27	0.42	44	15839	0.28	0.2	0.37
Acute and chronic thyroiditis	88	25310	0.35	0.28	0.43	45	15665	0.29	0.21	0.38
Diabetes mellitus, type 1	124	24894	0.50	0.42	0.59	65	15482	0.42	0.33	0.53
Diabetic ketoacidosis	40	25745	0.16	0.11	0.21	20	15891	0.13	0.08	0.19
Hypogonadism	≤10	n/a	0.02	0.01	0.05	≤10	n/a	0.01	0	0.03
Hypophysitis or hypopituitarism	90	25653	0.35	0.28	0.43	49	15826	0.31	0.23	0.41
Hypothyroidism	636	18796	3.38	3.13	3.65	325	12002	2.71	2.43	3.01
Thyroid hyperfunction disorders	121	25084	0.48	0.4	0.57	59	15554	0.38	0.29	0.49
Other safety events										
Abdominal pain	1825	5206	35.06	33.48	36.69	859	3098	27.73	25.92	29.63
Anemia	2925	11827	24.73	23.85	25.64	1,978	7576	26.11	24.98	27.28
Anorexia	649	24889	2.61	2.41	2.81	469	15316	3.06	2.79	3.35
Autoimmune disorder	182	25347	0.72	0.62	0.83	135	15657	0.86	0.73	1.02
Backache	1632	20754	7.86	7.49	8.25	963	13124	7.34	6.89	7.81
Constipation	2086	18379	11.35	10.87	11.85	1,404	11460	12.25	11.62	12.91
Cough	1421	19219	7.39	7.02	7.79	848	12341	6.87	6.42	7.35
Diarrhea	1502	20365	7.38	7.01	7.76	1,028	12630	8.14	7.65	8.65
Disorders of bilirubin excretion	66	25798	0.26	0.2	0.32	52	15904	0.33	0.25	0.43
Disorders of phosphorus metabolism	294	25265	1.16	1.04	1.3	177	15637	1.13	0.97	1.31
Dizziness and giddiness	977	21121	4.63	4.34	4.92	604	13504	4.47	4.13	4.84
Edema	1773	20466	8.66	8.27	9.07	1,107	12978	8.53	8.04	9.04
Encephalitis	18	25811	0.07	0.04	0.11	13	15904	0.08	0.05	0.14
Fever	1848	20676	8.94	8.54	9.35	1,313	12860	10.21	9.67	10.77
Guillain-Barre Syndrome	≤10	n/a	0.02	0.01	0.05	≤10	n/a	0.03	0.01	0.06
Hypertension	1243	10422	11.93	11.28	12.6	775	6820	11.36	10.58	12.18
Hypopotassemia	1808	20815	8.69	8.29	9.09	1,211	12983	9.33	8.81	9.86
Hyposmolality and/or hyponatremia	1475	23036	6.40	6.08	6.74	965	14302	6.75	6.33	7.18
Hypoxemia	1034	23624	4.38	4.12	4.65	590	14805	3.99	3.67	4.32
Iritis	36	25653	0.14	0.1	0.19	13	15839	0.08	0.05	0.14
Leukocytosis	1280	23275	5.50	5.2	5.81	792	14379	5.51	5.13	5.9
Localized superficial swelling, mass, or lump	385	25036	1.54	1.39	1.7	240	15436	1.55	1.37	1.76
Lymphocytopenia	17	25841	0.07	0.04	0.1	12	15926	0.08	0.04	0.13
Malaise and fatigue	2945	14296	20.60	19.87	21.35	2,089	9143	22.85	21.88	23.84
Myasthenia gravis	≤10	n/a	0.03	0.01	0.05	≤10	n/a	0.02	0.01	0.05
Myocarditis	≤10	n/a	0.01	0	0.02	≤10	n/a	0.01	0	0.04
Myositis	352	22731	1.55	1.39	1.72	192	14275	1.34	1.16	1.55
Nausea and vomiting	3436	13816	24.87	24.05	25.71	2,615	8033	32.55	31.32	33.82
Pain in joint	1584	14017	11.30	10.75	11.87	930	9254	10.05	9.42	10.71
Pain in limb	1741	16749	10.39	9.91	10.89	1,048	10874	9.64	9.07	10.24
Pancreatitis (acute or autoimmune)	146	25386	0.58	0.49	0.67	82	15678	0.52	0.42	0.65
Psoriasis	59	25370	0.23	0.18	0.3	30	15634	0.19	0.13	0.27
Respiratory abnormalities	≤10	n/a	0.01	0	0.02	≤10	n/a	0.01	0	0.03
Rheumatoid Arthritis	130	24912	0.52	0.44	0.62	70	15387	0.45	0.36	0.57
Sarcoidosis	32	25689	0.12	0.09	0.17	≤10	n/a	0.06	0.03	0.1
Systemic inflammatory response syndrome	116	25634	0.45	0.38	0.54	82	15789	0.52	0.42	0.64
Thrombocytopenia	1389	23210	5.98	5.68	6.31	1,181	13985	8.44	7.97	8.94
Uveitis	≤10	n/a	0.00	0	0.02	0	15938	0.00	0	0.02
Vitiligo	≤10	n/a	0.03	0.01	0.05	≤10	n/a	0.04	0.02	0.08
Overall Mortality	3051	18414	16.57	15.99	17.16	1903	8731	21.80	20.83	22.79

*Abbreviations*: *CI* Confidence interval, *IR* Incidence rate

^a^Estimates of IR are shown per 100 person-years. Incidence is calculated as the number of new events divided by the sum of person-time at risk, defined as the time between the start of follow-up and the date of the event. In each row, individuals who had a diagnosis of the applicable event prior to the start of follow-up (i.e., prevalent cases) were not included.

^b^Because serious infections were defined based on the need for acute care, all events occurred in hospital or emergency room settings.

Of the 25,868 person-years of follow-up in the advanced ovarian cancer cohort, 15,938 person-years (62%) were after a systemic anti-cancer therapy. When restricting to time after anti-cancer therapy, rates of many HOIs were similar compared to rates after the advanced stage index date, which included pre and post anti-cancer treatment time (e.g., any rash - after advanced stage: 3.0 per 100 person-years, after anti-cancer therapy: 3.1 person-years; renal failure – after advanced stage: 9.6 per 100 person-years, after anti-cancer therapy: 10.2 per 100 person-years; Table 3). However, incidence rates of some HOIs, such as serious infections, nausea and vomiting, malaise and fatigue, and thrombocytopenia, were higher after treatment (Table 3).

When restricting to severe HOIs occurring as the primary discharge diagnosis in inpatient or emergency room facilities, the incidence rates of all events were lower than overall HOI event rates, especially events such as nausea and vomiting, anemia, malaise/fatigue, and constipation, which declined over three-fold compared to the overall incidence rate (Table 3, Supplemental Table 4). Serious infections, abdominal pain, and renal failure were some of the most common hospitalized events noted as the primary discharge diagnosis (each > 4 per 100 person-years; Supplemental Table 5).

### Mortality, TTD, and TTNT analyses

In this cohort of 12,659 incident advanced stage ovarian cancer patients, 8374 patients were eligible to be linked to the NDI and thus available for the mortality analyses (66.2% of incident ovarian cancer cases). Characteristics between these patients and those who could not be linked to the NDI were largely similar except patients eligible for NDI linkage were older (median age 64 vs. 58) and had a less recent advanced stage index date; Supplemental Table 6).

**Fig. 1:**
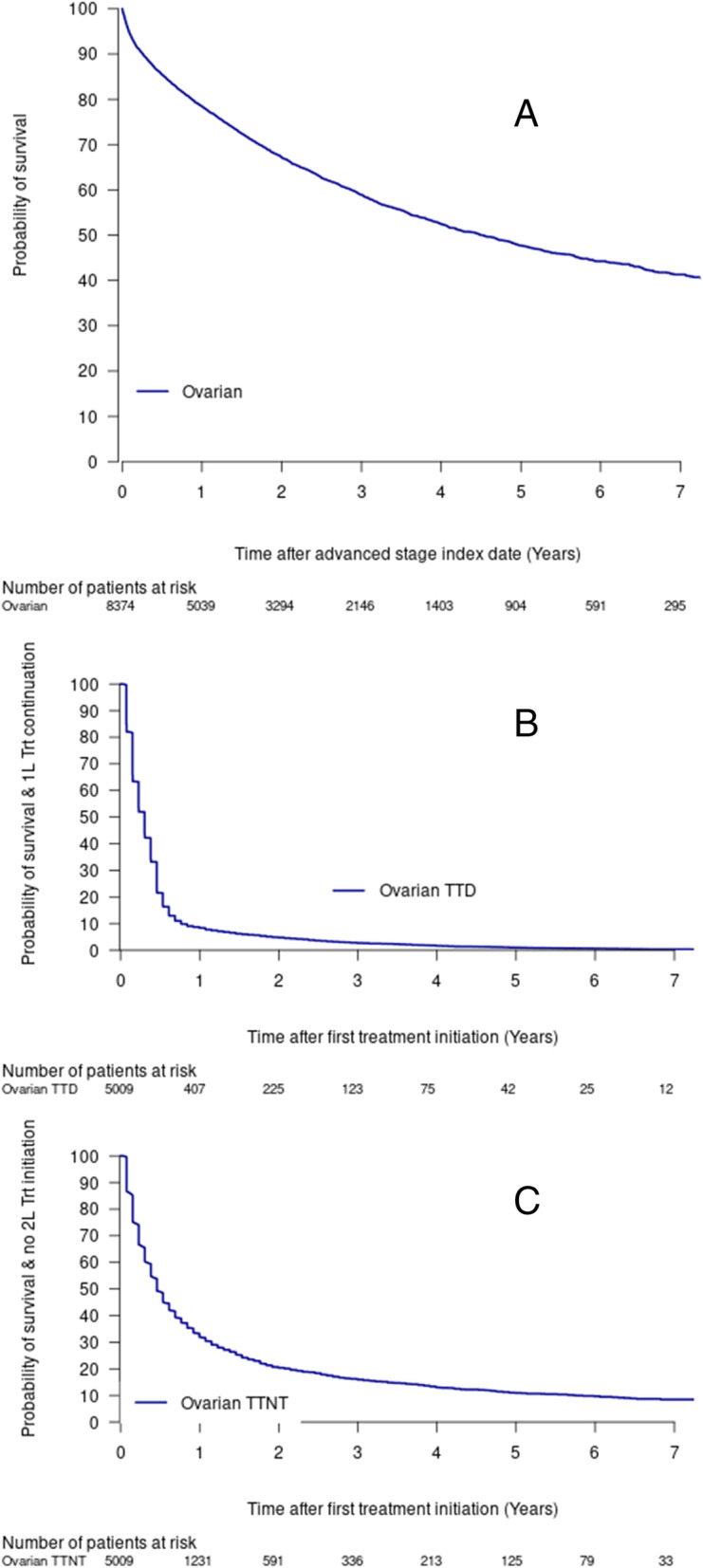
Advanced stage ovarian cancer, overall survival (**a**), time to treatment discontinuation or death (TTD) (**b**), time to next treatment or death (TTNT) (**c**). Abbreviations: 1 L, 1st Line; 2 L: 2nd Line; Trt, Treatment; TTD, treatment discontinuation or death; TTNT, time to next treatment or death

The median overall survival in this cohort was 4.5 years (95%CI = 4.17, 4.86; Fig. 1, Table 4). Approximately 25% of the cohort had died within 1.28 years (95%CI = 1.20, 1.37; Fig. 1), and the five-year survival was 47.7% (95%CI = 0.462–0.493; Table 4). Survival results were similar when excluding data after June 30, 2019 (five-year survival = 46.8% (95%CI = 0.451–0.484; Supplemental Table 7).

**Table 4:** Ovarian cancer overall survival, TTD, and TTNT estimates

	Estimate	95% CI	N of events	N remaining at risk
**Total number of events**	n/a	n/a	3051	5323
**Median OS (years)**	4.496	(4.172- 4.862)	n/a	n/a
**1-year survival rate**	78.4%	(77.5% - 79.3%)	1648	5032
**3-year survival rate**	58.9%	(57.6% - 60.2%)	2643	2120
**5-year survival rate**	47.7%	(46.2% - 49.3%)	2951	885
**Advanced stage ovarian cancer cohort, TTD**				
**Total number of events**	n/a	n/a	4951	58
**Median TTE (years)**	0.304	---*	n/a	n/a
**1-year "survival" rate**	8.3%	(7.6%, 9.1%)	4561	407
**Advanced stage ovarian cancer cohort, TTNT**				
**Total number of events**	n/a	n/a	3762	1247
**Median TTE (years)**	0.46	(0.460, 0.526)	n/a	n/a
**1-year "survival" rate**	31.9%	(30.6%, 33.3%)	3152	1231

*Abbreviations*: *TTD* Time to treatment discontinuation, *TTNT* Time to next treatment, *CI* Confidence interval, *N* Number, *TTE* Time to event, *OS* Overall survival

n/a Not applicable

*was not evaluable

The TTD and TTNT estimates among treated patients were lower than overall survival estimates with approximately half of the treated cohort having a treatment discontinuation or death within the first 4 months (Fig. 1, Table 4), or a second line treatment or death by about 6 months (0.46 years, 95%CI = 0.46, 0.53; Fig. 1, Table 4).

After NDI linkage, few fatal HOI events were identified, with hypertension, serious infections, and renal failure being the most common (data available upon request).

## Discussion

This study identified a large cohort of incident advanced stage ovarian cancer patients in US administrative claims and examined descriptive data on demographics, treatment patterns, safety events, and mortality rates. Incidence rates of serious infections, and symptoms such as abdominal pain, malaise and fatigue, and nausea and vomiting were high. Incidence rates of HOIs could be used as comparator rates for safety signals to help inform and contextualize the safety of new or future therapies for advanced stage ovarian cancer, especially for uncontrolled clinical trials. Our study, which used our previously validated predictive model for advanced stage ovarian cancer, [11] provides detailed information on the routine care of advanced stage ovarian cancer. In this population, over one-third of individuals received an ovarian-related surgery and over two-thirds of individuals received radiotherapy or systemic anti-cancer therapy during follow-up (i.e. after their advanced stage index date). Surgeries and treatments may have occurred prior to this advanced stage date (e.g. when they had early stage ovarian cancer or just before the index date), or after they have dropped out of the study (e.g. due to health plan discontinuation) as no minimal follow-up time was required.

The most commonly used treatments were chemotherapies such as alkylating agents and mitotic inhibitors, particularly in the first and second line of therapy. Other treatments such as antimetabolites and hormonal agents were more common in later lines of therapy. This cohort included some patients who had multiple malignances, and as diagnoses are not linked to a specific prescription, some of the included treatments may represent treatment for diseases outside of ovarian cancer. In addition, the treatment line algorithm may have some level of misclassification, as the results represent the treatment lines since the model estimated date of advanced cancer. Thus, some of the treatments noted in the first line could have been used in an adjuvant setting.

This study observed high incidence rates of certain HOIs during follow-up such as anemia, diarrhea, hypertension and fatigue that have been noted as adverse events in trials [4–6] and other smaller observational studies [19, 20]. This study also provides incidence rates of less common immune and endocrine-related events that have been unable to be robustly evaluated in previous studies given their limited sample size. While each of the 61 pre-specified HOI events did occur in at least one patient in this cohort, most of the immune and endocrine events were rare in advanced stage ovarian cancer patients, but events such as colitis and hypothyroidism were more common with incidence rates over three per 100 person-years of observation. The incidence of colitis and hypothyroidism in these women was not significantly higher after systemic therapy (Table 3). While, it is known that treatments such as platinum chemotherapy are associated with adverse events that impact quality of life, few studies have examined the occurrence of adverse events occurring among advanced stage ovarian cancer patients in a large real-world population. This is partially due to the lack of clinical stage information readily available in administrative claims. This study tried to provide proxies for such data through the incidence of HOIs among an advanced stage cancer population.

The HOIs in our study were not validated and it is expected that accuracy varies by safety event. In claims research, diagnosis, procedure, and prescription dispensing codes are used to reconstruct patients’ medical histories. As such, claims diagnoses are subject to misclassification and incidence estimates can vary widely based on the case definition used – a rate based on a definition that is very sensitive but not specific may be an overestimate, while a rate based on a definition that is specific but poorly sensitive may be an underestimate [21]. This is particularly relevant given that some of the outcomes used in the current study are based on clinical characteristics that are less likely to be assigned a diagnosis code (e.g., nausea, fatigue), and therefore would be captured in a claims database with poor sensitivity. These HOI algorithms would not capture fatal safety events if they occurred outside the healthcare system, although our linkage to the NDI could detect fatal HOIs, suggesting that HOIs were rarely noted on death certificates.

Survival of advanced stage ovarian cancer patients, while still relatively low, has been improving over time potentially due to the increasing number of therapeutic options. This study is also the first to our knowledge to provide estimates of TTD and TTNT (previously used as surrogates of disease progression during treatment) [22–24] for advanced stage ovarian cancer patients, in addition to overall survival. These proxies have been examined in other cancers and are correlated with progression free survival [16, 22]. In our study, we observe near ubiquitous treatment discontinuation (TTD) and transfer to second line (or later) therapies (TTNT) within a few months of initiation of the first line therapy for advanced disease, and while overall survival was longer than the TTD and TTNT measures, it was still poor with approximately half the patients dying within 5 years. We found that almost all patients with advanced stage ovarian cancer (> 95%) were diagnosed at an advanced stage, rather than progressing from an earlier stage. This may be an indication of a lack of screening for this disease suggesting that symptoms may be initially mistaken for other diseases or are not present until later in the disease progression, which could contribute to accelerated mortality. Recent trials suggest that the use of PARP inhibitors (e.g., veliparib and olaparib) alone or in combination with chemotherapy or VEGF inhibitors significantly improves progression-free survival in first-line, as maintenance therapy and after first-line platinum exposure in ovarian cancer [25–27]. If these findings are confirmed through a benefit in overall survival, these new treatment strategies will likely reshape the treatment landscape of the disease in the coming years with widespread use and likely improve the outcomes currently observed in this patient population.

Our cohort included both stage III and IV tumors among commercial insured US patients. This population is likely younger and with a higher social economic status than the general US ovarian cancer population, given our limited data on Medicare (> 65 year old) population and the lack of Medicaid data. The median overall survival, which was evaluated in a subset of population that was older than our overall population, was 4.5 years. In contrast, the 5-year survival rates based on Surveillance, Epidemiology, and End Results (SEER) data (US cancer registry) were 74% for regional tumors (spread to regional lymph nodes) at diagnosis and 29% for distant tumors (i.e. metastasized) (46% at 3 years) [28] suggesting overall survival may be modestly higher in our sample compared to SEER data assuming our sample largely is composed of distant stage cancers. While this difference could be related to age and higher income of our sample, there are also other explanations. For example, the start of follow-up time for SEER is the date of cancer diagnosis while in this study it is the date a patient has met the threshold of advanced stage cancer. Additionally, there could be imperfect sensitivity of NDI linkage for mortality, which would bias mortality rates downward. Published literature suggests NDI has a high sensitivity (97%) [29]. However, the sensitivity could be lower in patients with incomplete identifying information (e.g., missing social security number) which is present on at least a small subset of the HIRD.

In our main survival analyses, we censored a patient’s follow-up at the time they lost healthcare coverage eligibility in the HIRD (e.g., changed insurance plans). Some patients may leave their workplace and their related health plan as the disease progresses and deaths could occur at a differential rate – relatively soon after discontinuation of the health plan. To examine this possibility, we conducted an additional analysis where we did not censor at the discontinuation of the health plan. When using all available NDI mortality data, we found that the survival for ovarian cancer was similar to when censoring at health plan discontinuation (data available upon request) – suggesting that informed censoring was not a major source of bias.

## Conclusions

This study of over ten thousand advanced stage ovarian cancer patients in the US from 2010 to 2018 provides a description of the diverse treatment patterns, numerous HOIs, and relatively short survival time for these women. These data on incidence rates of HOIs could be utilized as comparator rates of safety events for new and future ovarian cancer therapies indicated for advanced stage ovarian cancer, which will be of particularly importance given the numerous new treatment options, such as PARP inhibitors, and increasing survival of this population.

## Supplementary information

**Additional file 1: Figure S1.** Example of a hypothetical patient “A” progressing from early to advanced stage ovarian cancer and definition of index date. Most patients (96.7%) in our cohort were classed as advanced stage at diagnosis. This hypothetical example would have been classified in those who “progressed from early to advanced stage ovarian cancer”, which represented 3.3% of patients in the cohort.

**Additional file 2: Table S1.** Codes used to define ovarian cancer. **Table S2.** Probability of advanced stage ovarian cancer for hypothetical patient “A” over time in the HIRD used to identify their index date. **Table S3.** Top 25 most common prescribed medication among 12,659 advanced stage ovarian cancer patients, 12 months before and after their advanced stage ovarian cancer date. **Table S4.** Advanced stage ovarian cancer cohort, cancer treatment received on or after the advanced stage date (*N* = 12,659). **Table S5.** Advanced stage ovarian cancer cohort, hospital or emergency room incidence rates of selected health outcomes of interest. **Table S6.** Characteristics by National Death Index (NDI) linkable status. **Table S7**. Ovarian cancer overall survival, excluding last 6 months of follow-up (July–December 2017).

## Data Availability

Data and further materials for this manuscript cannot be shared given privacy regulations.
