# Supplementary figures and images for: A real-world study on characteristics, treatments and outcomes in US patients with advanced stage ovarian cancer
Source: J Ovarian Res. 2020 Aug 31;13:101. doi: 10.1186/s13048-020-00691-y (PMC7461260; doi:10.1186/s13048-020-00691-y)

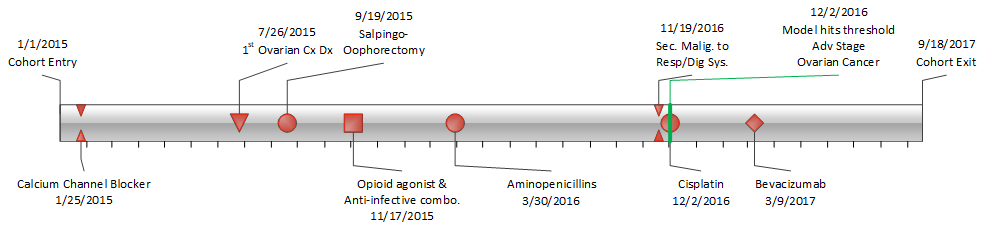

Supplement: Supplementary file 1 — Additional file 1: Figure S1. Example of a hypothetical patient “A” progressing from early to advanced stage ovarian cancer and definition of index date. Most patients (96.7%) in our cohort were classed as advanced stage at diagnosis. This hypothetical example would have been classified in those who “progressed from early to advanced stage ovarian cancer”, which represented 3.3% of patients in the cohort. [file 13048_2020_691_MOESM1_ESM.tif]
