# Supplementary material for: A real-world study on characteristics, treatments and outcomes in US patients with advanced stage ovarian cancer
Source: J Ovarian Res. 2020 Aug 31;13:101. doi: 10.1186/s13048-020-00691-y (PMC7461260; doi:10.1186/s13048-020-00691-y)
Supplement: Supplementary file 2 — Additional file 2: Table S1. Codes used to define ovarian cancer. Table S2. Probability of advanced stage ovarian cancer for hypothetical patient “A” over time in the HIRD used to identify their index date. Table S3. Top 25 most common prescribed medication among 12,659 advanced stage ovarian cancer patients, 12 months before and after their advanced stage ovarian cancer date. Table S4. Advanced stage ovarian cancer cohort, cancer treatment received on or after the advanced stage date (N = 12,659). Table S5. Advanced stage ovarian cancer cohort, hospital or emergency room incidence rates of selected health outcomes of interest. Table S6. Characteristics by National Death Index (NDI) linkable status. Table S7. Ovarian cancer overall survival, excluding last 6 months of follow-up (July–December 2017). [file 13048_2020_691_MOESM2_ESM.docx]

**Supplemental Table 1: Codes used to define ovarian cancer^1^**

| **Code** | **Type** | **Description** |
| --- | --- | --- |
| 183.0 | ICD-9 | Malignant neoplasm of ovary |
| C56 | ICD-10 | Malignant neoplasm of ovary |
| C56.1 | ICD-10 | Malignant neoplasm of right ovary |
| C56.2 | ICD-10 | Malignant neoplasm of left ovary |
| C56.9 | ICD-10 | Malignant neoplasm of unspecified ovary |

1: Participants included in this study had advanced stage ovarian cancer, the advanced stage classification was defined through information available in a cancer registry, HIRE oncology, or met the criteria for the predictive model algorithm for advanced ovarian cancer. Further described in Esposito et al. (1)

**Supplemental Table 2. Probability of advanced stage ovarian cancer for hypothetical patient “A” over time in the HIRD used to identify their index date**

|  | | | **Advanced Subscore** | |  |
| --- | --- | --- | --- | --- | --- |
| **ID** | **Evaluation Date** | **Criteria Name** | **Subscore^1^** | **Sum of Subscores** | **Predicted Probability of Advanced Stage Ovarian Cancer** |
|  |  | (intercept) |  | -0.96 |  |
| A | 1/25/2015 | Calcium channel blocker | -0.07 | -1.03 | 26.3% |
| A | 7/26/2015 | 1^st^ ovarian cancer diagnosis code | *reference* |  |  |
| A | 9/19/2015 | Salpingo-Oophorectomy | 0.08 | -0.95 | 27.9% |
| A | 11/19/2015 | Opioid agonist | 0.10 | -0.85 | 29.9% |
| A | 11/19/2015 | Anti-infective combinations | 0.14 | -0.71 | 33.0% |
| A | 3/30/2016 | Aminopenicillins | 0.03 | -0.68 | 33.6% |
| A | 12/2/2016 | Secondary Malig: Resp/Dig. System | 1.47 | 0.79 | 68.8% |
| **A** | **12/2/2016** | **Cisplatin - Advanced_indexdate**^2^ | **0.60** | **1.39** | **80.0%** |
| A | 3/9/2017 | Bevacizumab | 1.01 | 2.4 | 91.6% |

1: Coefficient from predictive model algorithm for advanced stage ovarian cancer

2: The hypothetical patient in the table above was defined as having advanced stage ovarian cancer when the model reached the threshold of 80% probability that the patient has advanced stage ovarian cancer on 12/2/2016 when they initiated cisplatin. This date is their advanced stage index date.

**Supplemental Table 3. Top 25 most common prescribed medication among 12,659 advanced stage ovarian cancer patients, 12 months before and after their advanced stage ovarian cancer date^1,2^**

| **12 months prior to advanced stage date** | | | |
| --- | --- | --- | --- |
| GPI Code | Description | N | % |
| 6599 | *Opioid Combinations** | 5263 | 41.58 |
| 0500 | *Fluoroquinolones** | 3349 | 26.46 |
| 2210 | *Glucocorticosteroids** | 3146 | 24.85 |
| 3940 | *HMG CoA Reductase Inhibitors** | 3015 | 23.82 |
| 5710 | *Benzodiazepines** | 2960 | 23.38 |
| 4927 | *Proton Pump Inhibitors** | 2570 | 20.30 |
| 5025 | *5-HT3 Receptor Antagonists** | 2492 | 19.69 |
| 6610 | *Nonsteroidal Anti-inflammatory Agents (NSAIDs)** | 2417 | 19.09 |
| 6510 | *Opioid Agonists** | 2310 | 18.25 |
| 0340 | *Azithromycin** | 2109 | 16.66 |
| 2810 | *Thyroid Hormones** | 1982 | 15.66 |
| 5816 | *Selective Serotonin Reuptake Inhibitors (SSRIs)** | 1943 | 15.35 |
| 3320 | *Beta Blockers Cardio-Selective** | 1913 | 15.11 |
| 5920 | *Phenothiazines** | 1751 | 13.83 |
| 3610 | *ACE Inhibitors** | 1614 | 12.75 |
| 3400 | *Calcium Channel Blockers** | 1505 | 11.89 |
| 4420 | *Sympathomimetics** | 1487 | 11.75 |
| 4699 | *Laxative Combinations** | 1425 | 11.26 |
| 0210 | *Cephalosporins - 1st Generation** | 1398 | 11.04 |
| 6020 | *Non-Barbiturate Hypnotics** | 1384 | 10.93 |
| 7260 | *Anticonvulsants - Misc.** | 1356 | 10.71 |
| 0120 | *Aminopenicillins** | 1328 | 10.49 |
| 1699 | *Anti-infective Misc. - Combinations** | 1283 | 10.14 |
| 3699 | *Antihypertensive Combinations** | 1248 | 9.86 |
| 7970 | *Potassium** | 1219 | 9.63 |
| **12 months after advanced stage date** | | | |
| GPI Code | Description | N | % |
| 6599 | *Opioid Combinations** | 5863 | 46.31 |
| 5025 | *5-HT3 Receptor Antagonists** | 4692 | 37.06 |
| 2210 | *Glucocorticosteroids** | 4371 | 34.53 |
| 5710 | *Benzodiazepines** | 3897 | 30.78 |
| 6510 | *Opioid Agonists** | 3636 | 28.72 |
| 0500 | *Fluoroquinolones** | 3434 | 27.13 |
| 5920 | *Phenothiazines** | 3414 | 26.97 |
| 4927 | *Proton Pump Inhibitors** | 2948 | 23.29 |
| 3940 | *HMG CoA Reductase Inhibitors** | 2414 | 19.07 |
| 6610 | *Nonsteroidal Anti-inflammatory Agents (NSAIDs)** | 2385 | 18.84 |
| 8310 | *Heparins And Heparinoid-Like Agents** | 2158 | 17.05 |
| 5816 | *Selective Serotonin Reuptake Inhibitors (SSRIs)** | 2107 | 16.64 |
| 3320 | *Beta Blockers Cardio-Selective** | 2015 | 15.92 |
| 7260 | *Anticonvulsants - Misc.** | 1950 | 15.40 |
| 2810 | *Thyroid Hormones** | 1898 | 14.99 |
| 9085 | *Local Anesthetics - Topical** | 1860 | 14.69 |
| 7970 | *Potassium** | 1835 | 14.50 |
| 0340 | *Azithromycin** | 1719 | 13.58 |
| 0210 | *Cephalosporins - 1st Generation** | 1662 | 13.13 |
| 6020 | *Non-Barbiturate Hypnotics** | 1662 | 13.13 |
| 3720 | *Loop Diuretics** | 1472 | 11.63 |
| 3400 | *Calcium Channel Blockers** | 1463 | 11.56 |
| 1699 | *Anti-infective Misc. - Combinations** | 1436 | 11.34 |
| 3610 | *ACE Inhibitors** | 1379 | 10.89 |
| 4420 | *Sympathomimetics** | 1241 | 9.80 |

Abbreviations: N - number, GPI - generic product identifier, HMG CoA - 3-hydroxy-3-methyl-glutaryl-coenzyme A, ICD-9-CM - International Classification of Diseases, Ninth Revision, Clinical Modification, Misc. - miscellaneous

1: The cohort includes patients who had at least one ICD-9-CM or ICD-10 diagnosis code for ovarian cancer, were continuously enrolled in a health plan contributing data to the HIRD for at least six months, and were confirmed to have advanced ovarian cancer based on staging information from either a cancer registry or the HIRE Oncology data or met the predictive model algorithm for advanced stage ovarian cancer.

2: Medication subclasses defined by the first four digits of the GPI code were ranked based on the number of cohort members with at least one pharmacy dispensing of a medication included in the subclass. Medications administered in a healthcare setting are not captured in this classification.

**Supplemental Table 4. Advanced stage ovarian cancer cohort, cancer treatment received on or after the advanced stage date (N=12,659)^1,2^**

| **Treatment** | **Among all incident cancer patients** | |
| --- | --- | --- |
| At least one surgical procedure (N, %) | N (%) | 5,131 (40.53%) |
| Oophorectomy (N, %) | N (%) | 896 (7.08%) |
| Salpingo-oophorectomy (N, %) | N (%) | 3,450 (27.25%) |
| Total abdominal hysterectomy and bilateral salpingo-oophorectomy (N, %) | N (%) | 2,817 (22.25%) |
| Cytoreductive/debulking surgery | N (%) | 2,516 (19.88%) |
| Palliative surgery for relief of small bowel obstruction (N, %) | N (%) | 4,416 (34.88%) |
| Number of computed tomography (CT) scans per month | Mean (SD) | 0.47 (0.88) |
|  | Q1 | 0.06 |
|  | Median | 0.31 |
|  | Q3 | 0.62 |
| Among those with at least one surgical procedure |  |  |
| Chemotherapy administered before surgical procedure (N, %) | N (%) | 1,223 (23.84%) |
| Chemotherapy administered after surgical procedure (N, %) | N (%) | 3,633 (70.80%) |
| Radiation therapy (N, %) | N (%) | 1,030 (8.14%) |
| Systemic anti-cancer therapy (not including radiotherapy) (N, %)^3^ | N (%) | 7,723 (61.01%) |
| Ovarian cancer platinum status |  |  |
| Platinum sensitive | N (%) | 934 (12.09%) |
| Platinum resistant | N (%) | 1,181 (15.29%) |
| Platinum refractory | N (%) | 3,220 (41.69%) |
| Unknown | N (%) | 2,388 (30.92%) |
| Number of administrations identified | Mean (SD) | 20.11 (21.94) |
|  | Q1 | 6 |
|  | Median | 14 |
|  | Q3 | 27 |
| Duration of anti-cancer therapy (days) | Mean (SD) | 292.75 (317.96) |
|  | Q1 | 101 |
|  | Median | 175 |
|  | Q3 | 376 |
| Anti-cancer therapy restarted after more than six months without an administration (N, %) |  | 2,696 (34.91%) |
| Number of treatment lines observed ^3^ | N (%) | 12159 (96.05%) |
|  | Mean (SD) | 2.11 (2.68) |
|  | Q1 | 0 |
|  | Median | 1 |
|  | Q3 | 3 |
| **Highest treatment line observed** |  |  |
| No treatment lines | N (%) | 3834 (31.53%) |
| First line | N (%) | 2990 (24.59%) |
| Second line | N (%) | 1624 (13.36%) |
| Third line | N (%) | 1224 (10.07%) |
| Fourth line or higher | N (%) | 2487 (20.45%) |

Abbreviation: N - number, CT - computed tomography

1: The cohort includes patients who had at least one ICD-9-CM or ICD-10 diagnosis code for ovarian cancer, were continuously enrolled in a health plan contributing data to the HIRD for at least six months, and were confirmed to have advanced ovarian cancer based on staging information from either a cancer registry or the HIRE Oncology data or met the predictive model algorithm for advanced stage ovarian cancer.

2: Proportions based on total number of incident cases.

3: Patients with follow-up periods shorter than 28 days (specified segment length) were excluded from the treatment line related analyses.

**Supplemental Table 5. Advanced stage ovarian cancer cohort, hospital or emergency room incidence rates of selected health outcomes of interest**

| Health outcome of interest | **Hospitalization or Emergency Room** | | | | |
| --- | --- | --- | --- | --- | --- |
|  | Events | Person-years | IR^1^ | 95% CI | |
| Serious infection^3^ | 6,662 | 25868 | 25.78 | 25.17 | 26.41 |
| Rash |  |  |  |  |  |
| Any Rash | 106 | 25429 | 0.42 | 0.34 | 0.5 |
| Severe cutaneous rash safety events | 21 | 25781 | 0.08 | 0.05 | 0.12 |
| Colitis | 388 | 24639 | 1.57 | 1.42 | 1.74 |
| Pneumonitis |  |  |  |  |  |
| Interstitial lung disease | 42 | 25767 | 0.16 | 0.12 | 0.22 |
| Hypersensitivity pneumonitis | ≤10 | n/a | 0.02 | 0.01 | 0.04 |
| Pneumonitis or acute interstitial pneumonitis | 54 | 25764 | 0.21 | 0.16 | 0.27 |
| Hepatitis |  |  |  |  |  |
| Hepatic failure | 185 | 25411 | 0.73 | 0.63 | 0.84 |
| Autoimmune hepatitis | ≤10 | n/a | 0.00 | 0 | 0.02 |
| Hepatitis (not specified as viral) | 14 | 25832 | 0.05 | 0.03 | 0.09 |
| Liver disorder | 338 | 25181 | 1.34 | 1.2 | 1.49 |
| Transaminases increased | 38 | 25814 | 0.15 | 0.11 | 0.2 |
| Nephritis | 1,114 | 23974 | 4.65 | 4.38 | 4.93 |
| Renal failure | 1,124 | 24065 | 4.67 | 4.4 | 4.95 |
| Endocrinopathies |  |  |  |  |  |
| Adrenal insufficiency | 20 | 25823 | 0.08 | 0.05 | 0.12 |
| Acute and chronic thyroiditis | ≤10 | n/a | 0.01 | 0 | 0.02 |
| Diabetes mellitus, type 1 | 27 | 25738 | 0.10 | 0.07 | 0.15 |
| Diabetic ketoacidosis | 17 | 25808 | 0.07 | 0.04 | 0.1 |
| Hypogonadism | ≤10 | n/a | 0.00 | 0 | 0.02 |
| Hypophysitis or hypopituitarism | 22 | 25828 | 0.09 | 0.05 | 0.13 |
| Hypothyroidism | 105 | 25569 | 0.41 | 0.34 | 0.49 |
| Thyroid hyperfunction disorders | 14 | 25791 | 0.05 | 0.03 | 0.09 |
| Other safety events |  |  |  |  |  |
| Abdominal pain | 2,952 | 12345 | 23.91 | 23.06 | 24.79 |
| Anemia | 956 | 23385 | 4.09 | 3.84 | 4.35 |
| Anorexia | 57 | 25782 | 0.22 | 0.17 | 0.28 |
| Autoimmune disorder | 14 | 25827 | 0.05 | 0.03 | 0.09 |
| Backache | 444 | 24784 | 1.79 | 1.63 | 1.96 |
| Constipation | 713 | 24304 | 2.93 | 2.72 | 3.16 |
| Cough | 545 | 24365 | 2.24 | 2.05 | 2.43 |
| Diarrhea | 418 | 24825 | 1.68 | 1.53 | 1.85 |
| Disorders of bilirubin excretion | 17 | 25857 | 0.07 | 0.04 | 0.1 |
| Disorders of phosphorus metabolism | ≤10 | n/a | 0.03 | 0.02 | 0.06 |
| Dizziness and giddiness | 423 | 24254 | 1.74 | 1.58 | 1.92 |
| Edema | 673 | 24536 | 2.74 | 2.54 | 2.96 |
| Encephalitis | ≤10 | n/a | 0.03 | 0.02 | 0.06 |
| Fever | 1,184 | 22980 | 5.15 | 4.87 | 5.45 |
| Guillain-Barre Syndrome | ≤10 | n/a | 0.01 | 0 | 0.02 |
| Hypertension | 862 | 22596 | 3.81 | 3.57 | 4.08 |
| Hypopotassemia | 292 | 25239 | 1.16 | 1.03 | 1.3 |
| Hyposmolality and/or hyponatremia | 347 | 25319 | 1.37 | 1.23 | 1.52 |
| Hypoxemia | 509 | 24888 | 2.05 | 1.87 | 2.23 |
| Iritis | ≤10 | n/a | 0.01 | 0 | 0.02 |
| Leukocytosis | 350 | 25287 | 1.38 | 1.24 | 1.53 |
| Localized superficial swelling, mass, or lump | 126 | 25696 | 0.49 | 0.41 | 0.58 |
| Lymphocytopenia | ≤10 | n/a | 0.01 | 0 | 0.03 |
| Malaise and fatigue | 1,189 | 23563 | 5.05 | 4.77 | 5.34 |
| Myasthenia gravis | ≤10 | n/a | 0.01 | 0 | 0.02 |
| Myocarditis | 0 | 25865 | 0.00 | 0 | 0.01 |
| Myositis | 25 | 25771 | 0.10 | 0.06 | 0.14 |
| Nausea and Vomiting | 1,685 | 22249 | 7.57 | 7.22 | 7.94 |
| Pain in joint | 635 | 23267 | 2.73 | 2.52 | 2.95 |
| Pain in limb | 796 | 23256 | 3.42 | 3.19 | 3.67 |
| Pancreatitis (acute or autoimmune) | 91 | 25549 | 0.36 | 0.29 | 0.44 |
| Psoriasis | ≤10 | n/a | 0.01 | 0 | 0.03 |
| Respiratory abnormalities | ≤10 | n/a | 0.00 | 0 | 0.02 |
| Rheumatoid Arthritis | 11 | 25807 | 0.04 | 0.02 | 0.07 |
| Sarcoidosis | ≤10 | n/a | 0.03 | 0.01 | 0.06 |
| Systemic inflammatory response syndrome | 50 | 25772 | 0.19 | 0.15 | 0.25 |
| Thrombocytopenia | 215 | 25502 | 0.84 | 0.74 | 0.96 |
| Uveitis | 0 | 25864 | 0.00 | 0 | 0.01 |
| Vitiligo | 0 | 25868 | 0.00 | 0 | 0.01 |

Abbreviations: CI, confidence interval; IR, incidence rate

1: Estimates of IR are shown per 100 person-years. Incidence is calculated as the number of new events divided by the sum of person-time at risk, defined as the time between the start of follow-up and the date of the event. In each row, individuals who had a diagnosis of the applicable event prior to the start of follow-up (i.e., prevalent cases) were not included.

2: While most safety events were defined based on two or more applicable diagnosis codes in any setting for "any event" and at least one principal discharge diagnosis with an applicable code for "hospitalization or emergency room events," infusion related reactions were defined based on a single pre-specified algorithm that considered symptoms, time since an observed administration or dispensing, and place of service. Given the acute nature of these outcomes we did not exclude patients who had the outcome prior to study follow-up (i.e., prevalent cases during the baseline period) and counted each separate occurrence of the events as long as they were at least seven days after the occurrence of the prior event.

3: Because serious infections were defined based on the need for acute care, all events occurred in hospital or emergency room settings. Also given their acute nature, these outcomes did not exclude patients who had the outcome prior to study follow-up (i.e., prevalent cases during the baseline period).

**Supplemental Table 6. Characteristics by National Death Index (NDI) linkable status**

| **Characteristic** | | **NDI non-linkable Incident Cases** | **NDI linkable Incident Cases ^0^** |
| --- | --- | --- | --- |
| Total confirmed advanced incident cancer cases (N, %)^1,2^ | |  |  |
|  | Advanced stage at diagnosis, N (%)^3^ | 3,869 (96.94%) | 8,368 (96.54%) |
|  | Diagnosed as early stage and progressed to advanced stage, N (%) | 122 (3.06%) | 300 (3.46%) |
| Age (years) |  |  |  |
|  | Mean (SD) | 57.30 (12.80) | 64.08 (14.00) |
|  | Q1 | 50 | 55 |
|  | Median | 58 | 64 |
|  | Q3 | 64 | 74 |
| Region |  |  |  |
|  | Midwest | 801 (22.23%) | 2,635 (31.15%) |
|  | Northeast | 791 (21.95%) | 1,502 (17.75%) |
|  | South | 1,147 (31.83%) | 1,829 (21.62%) |
|  | West | 864 (23.98%) | 2,494 (29.48%) |
| Year of index date |  |  |  |
|  | 2010-2011 | 984 (24.66%) | 3,728 (43.01%) |
|  | 2012-2014 | 1,408 (35.28%) | 3,060 (35.29%) |
|  | 2015-2018 | 1,599 (40.07%) | 1,880 (21.69%) |
| Plan type |  |  |  |
|  | Commercial | 3,980 (99.72%) | 5,308 (61.24%) |
|  | Medicare Advantage | ≤10 | 1,507 (17.39%) |
|  | Medicare, Other | ≤10 | 1,853 (21.38%) |
| Duration in the HIRD prior to cancer diagnosis (months) | |  |  |
|  | Mean (SD) | 41.38 (27.46) | 51.29 (28.06) |
|  | Q1 | 18.23 | 27.07 |
|  | Median | 35.45 | 49.51 |
|  | Q3 | 57.36 | 72.71 |
| Duration of follow-up (months) | |  |  |
|  | Mean (SD) | 22.41 (20.57) | 25.49 (24.36) |
|  | Q1 | 6.77 | 6.28 |
|  | Median | 16.62 | 17.61 |
|  | Q3 | 31.72 | 36.9 |
| Deyo-Charlson Comorbidity Index (DCI) during follow-up | |  |  |
|  | Mean (SD) | 6.22 (2.58) | 6.39 (2.65) |
|  | Q1 | 4 | 5 |
|  | Median | 6 | 6 |
|  | Q3 | 8 | 8 |
| Count of distinct dispensed drugs used during follow-up | |  |  |
|  | Mean (SD) | 12.45 (9.66) | 12.68 (9.80) |
|  | Q1 | 5 | 5 |
|  | Median | 11 | 12 |
|  | Q3 | 18 | 18 |

Abbreviations: NDI- National Death Index, N - number, PPPY - per patient per year, SD - standard deviation, US - United States, Q - quartile, ED - emergency department.

0: NDI-linkable cases include all individuals who were fully insured by Anthem during the study period and were eligible to be sent to the NDI for mortality/survival related analyses. The survival analyses are restricted to these patients, and include all of these patients except for a small subset of patient who were fully insured by Anthem during the study period, but at some point in the past were part of an "administrative service only" plan.
1: The cohort includes patients who had at least one ICD-9-CM or ICD-10 diagnosis code for ovarian cancer, were continuously enrolled in a health plan contributing data to the HIRD for at least six months, and were confirmed to have advanced ovarian cancer based on staging information from either a cancer registry or the HIRE Oncology data or met the predictive model algorithm for advanced stage ovarian cancer.
2: Incident cases are individuals for whom at least six months of data were available in the HIRD prior to the first diagnosis of ovarian cancer in claims.
3: Cases are defined as "advanced stage at diagnosis" if their advanced stage date (from cancer registry, HIRE Oncology, or predictive model) was within one month of their first cancer diagnosis in claims, otherwise they are defined as "progressed from early to advanced stage".

**Supplemental Table 7. Ovarian cancer overall survival, excluding last six months of follow-up (July-December 2017)**

|  | **Estimate** | **95% CI** | **N of events** | **N remaining at risk** |
| --- | --- | --- | --- | --- |
| **Total number of events** | n/a | n/a | 2968 | 5165 |
| **Median OS (years)** | 4.282 | (4.049- 4.674) | n/a | n/a |
| **1-year survival rate** | 78.2% | 77.3%-79.2% | 1614 | 4840 |
| **3-year survival rate** | 58.2% | 56.9%-59.5% | 2586 | 1987 |
| **5-year survival rate** | 46.8% | 45.1%-48.4% | 2880 | 797 |

Abbreviations: CI, confidence interval; N, number; OS, overall survival.
